# Socioeconomic position and health status of people who live near busy roads: the Rome Longitudinal Study (RoLS)

**DOI:** 10.1186/1476-069X-9-41

**Published:** 2010-07-21

**Authors:** Giulia Cesaroni, Chiara Badaloni, Valeria Romano, Eugenio Donato, Carlo A Perucci, Francesco Forastiere

**Affiliations:** 1 Department of Epidemiology of the Regional Health Service, Lazio Region, via S.Costanza 53, 00198 Rome, Italy; 2 Department of Environmental Policies, Rome Local Council, Circonvallazione Ostiense 191, 00154 Rome, Italy

## Abstract

**Background:**

Subjects living close to high traffic roads (HTR) are more likely to suffer from air-pollution related morbidity and mortality. The issue has large public health consequences but few studies have described the main socio-demographic characteristics of people exposed to traffic.

**Objectives:**

To characterise a large cohort of residents in Rome according to different measures of traffic exposure, socioeconomic position (SEP), and baseline health status.

**Methods:**

Residents of Rome in October 2001 were selected. Individual and area-based SEP indices were available. GIS was used to obtain traffic indicators at residential addresses: distance from HTR (> = 10,000 vehicles/day), length of HTR, average daily traffic count, and traffic density within 150 meters of home. Hospitalisations in the 5-year period before enrolment were used to characterise health status. Logistic and linear regression analyses estimated the association between traffic exposure and socio-demographic characteristics.

**Results:**

We selected 1,898,898 subjects with complete SEP information and GIS traffic indicators. A total of 320,913 individuals (17%) lived within 50 meters of an HTR, and 14% lived between 50 and 100 meters. These proportions were higher among 75+ year-old subjects. Overall, all traffic indicators were directly associated with SEP, with people living in high or medium SEP areas or with a university degree more likely to be exposed to traffic than people living in low SEP areas or with a low level of education. However, an effect modification by area of residence within the city was seen and the association between traffic and SEP was reversed in the city centre.

**Conclusions:**

A large section of the population is exposed to traffic in Rome. Elderly people and those living in areas of high and medium SEP tend to be more exposed. These findings are related to the historical stratification of the population within the city according to age and socioeconomic status.

## Background

There is convincing scientific evidence that exposure to air pollution, in particular ambient particulate matter (PM), is related to both short and long-term health effects. Increased mortality and hospitalizations for cardiopulmonary causes have been noted in several studies evaluating the short-term effects of PM_10 _or PM_2.5 _[1]. Some longitudinal studies conducted in the US and in Europe did find a consistent association between long term exposure to air pollution and natural mortality, especially for cardiovascular diseases [2-7].

In urban areas a relevant source of air pollution is vehicular traffic as the importance of other sources (e.g. industry, power plants) is declining. In some of the studies evaluating health effects, proxy measures of exposure have been used, such as distance from busy roads, distance-weighted traffic density or the length of main street segments within a buffer from home [8-12]. These proxy measures tend to be correlated with other more objective measures (for example, NO_2 _concentrations estimated by dispersion modelling and/or land use regression models) and are relatively easy to obtain using Geographic Information System (GIS) [13]. Some studies have directly assessed the relationship between living along busy roads and mortality or morbidity. For example, Hoek and colleagues found, in a cohort of adults aged 55-69 years, that cardiopulmonary mortality was associated with living near a major road [5]. In the SAPALDIA (Swiss Cohort Study on Air Pollution and Lung Diseases in Adults) study an increased risk of regular phlegm and wheezing in non smokers living within 20 m of a main street was found [8]. All the literature has been recently reviewed by a scientific panel from the Health Effects Institute (HEI panel on the Health Effects of Traffic-Related Air Pollution) in the US [14]. The Panel concluded that the evidence was "sufficient" to infer a causal relationship between exposure to traffic and exacerbation of asthma and "suggestive but not sufficient" to infer a causal relationship with onset of childhood asthma, non-asthma respiratory symptoms, impaired lung function, and total and cardiovascular mortality.

When evaluating the relationship between living along busy roads and mortality/morbidity, the potential role of socioeconomic status should be considered. The relationship between socioeconomic position and exposure to air pollution has been extensively reviewed and "environmental justice" concerns have been raised [15-21]. In fact, low socioeconomic groups of the population seem to suffer from the worst environmental conditions, including poor air quality [18-24], and tend to be more vulnerable to air pollution [19,25-27]. However, the extent to which low socioeconomic position and proximity to busy roads are related is not well-defined.

Few studies have characterised subjects living near high traffic roads in urban areas to better understand the exposure characteristics and the susceptibility factors of air pollution. The lack of studies is somewhat surprising given the potential public heath impact of traffic exposure. We established a large cohort of people who were resident in Rome in 2001 with information on socio-demographic characteristics and baseline health status to study long-term effects of traffic-related air pollution. We wished to use diverse indices of socioeconomic position (both at the individual and neighbourhood level) and different geographic information system (GIS) measures to characterize the residents who live close to busy roads in Rome. In the present paper we describe the main baseline characteristics of the cohort and examine the association of exposure to traffic with socio-demographic characteristics.

## Methods

### Setting

Rome is the largest Italian city with a population of about 2.6 millions inhabitants on a surface of 1290 km^2^. It is a radiant city, and the most important roads are still the ancient roman roads that starting from the centre, the Roman Forum, connect the city with the rest of the country in all the directions. During the last century, the urban development in Rome took place gradually from the centre to the suburbs, with a higher population density in the centre compared to the periphery [28].

### Study Population

The cohort was defined from Rome Municipal Register's data. We enrolled all residents of Rome on the 21^st ^October 2001; data were available on gender, age, and residential history. Using a variety of record-linkages procedures, under strict control to protect individual privacy, we collected additional information for each study member. In particular, individual data from the 2001 Census were used for indices of socioeconomic position at the baseline. The 2001 residential addresses were used to estimate environmental exposures and traffic-related air pollution indices. Individual hospital admissions from public and private hospitals in Italy, during the period 1996 to 2001, were available to provide the morbidity history of the subjects.

### Area-based and individual information on the socioeconomic position

**Figure 1:**
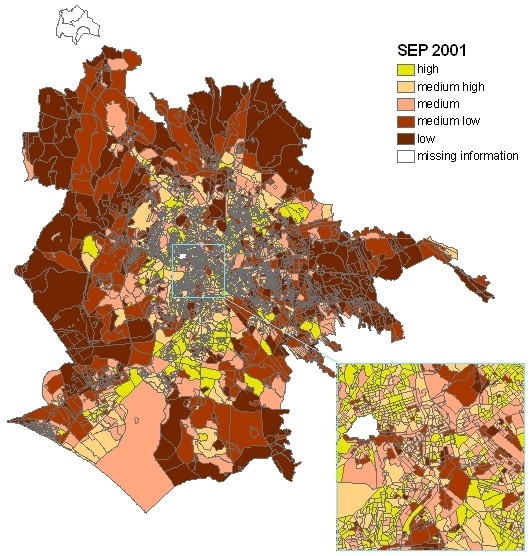
**Map of Rome by socioeconomic position (SEP)**.

A composite area-based index of socioeconomic position (SEP) by census block was built using the 2001 Census of Rome. Briefly, we used 4,888 census blocks with at least 50 inhabitants (average population: 500 subjects) as the units of observation. We considered census information that represented various socioeconomic parameters (occupation, education, housing tenure, family composition, and foreign status (yes or no)) and each census block was characterized. We performed a factor analysis to create a composite indicator, and we used the quintiles of its distribution in census blocks to obtain a 5-level area-based index [29]. To obtain the index for all census blocks of Rome, we assigned a SEP level to census blocks with fewer than 50 inhabitants (0.4% of the population) according to the levels of contiguous blocks. The area based SEP has been validated with individual census data [29] (for example in the highest category of area based SEP there was 29% of people with a university degree vs. 5% in the lowest category of SEP), the index is highly correlated with a small area income index based on 1998 Tax Register data [30], and it has been associated with overall and cause-specific mortality and incidence of specific diseases such as stroke [31,32]. Figure 1 shows the map of the city by SEP.

From the 2001 Census we obtained individual data on educational level (grouped into four categories: University, High school, Secondary school, Primary school), employment status (Employed, Looking for first employment, Unemployed, Student, Housewife, Retired, Military or civil service, Unable to work, Other), occupation (Non-manual: Managers, Highly-skilled, Medium-skilled, Unskilled; Manual: Services, Farmer, Highly-skilled, Medium-skilled, Unskilled; Military forces).

### Environmental characteristics at the residential address

*Geographical information system (GIS) *indices were developed for each individual. We geocoded each subject's residence as of 21^st ^October 2001 using the interpolation method within road segments. To locate the address on the map we used the Italian road network (Tele Atlas, Italy). The City Council of Rome provided the traffic data for all major roads in Rome as of 2005, i.e. 6,585 road segments which represented the 26% of all roads, and included the totality of roads with more than 10,000 vehicles per day (2,228 segments).

**Figure 2:**
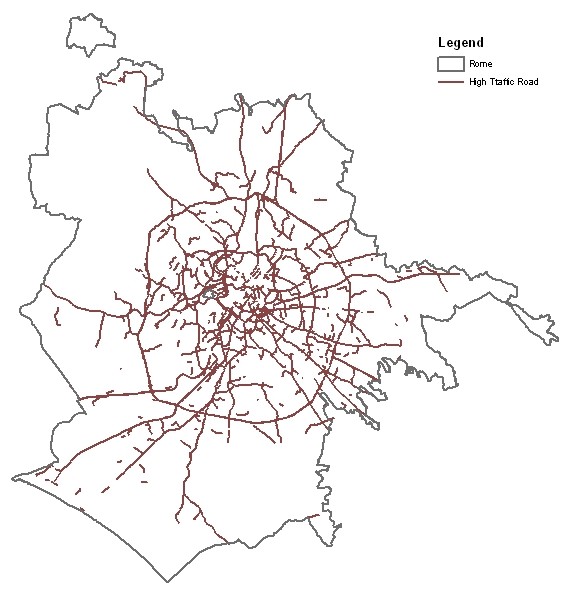
**Map of Rome with high traffic roads (HTR, > = 10,000 vehicles/day)**.

We defined as high traffic (HTR) roads all road segments where at least 10,000 vehicles travelled per day. Figure 2 shows the map of Rome with the HTRs. We defined different GIS indicators for each residential address: the distance from the residence to the nearest HTR, the total length of the HTR segments within a 150 m buffer zone, the daily average traffic counts from the closest HTR within 150 m, and the traffic density within 150 m. The latter was defined as the sum of the products of each HTR segment length by the estimated annual average daily traffic count of the HTR segment (within the 150 meter buffer zone around the residence address) [10], divided by the area of the buffer:

We also defined a categorical variable of traffic density within 150 m as the quartiles of the distribution of the continuous variable.

Similar to the SAPALDIA study [8], we applied buffers of different radii (50, 100, 150, and 250 m) to the residences and intersected the buffers with the list of high traffic roads to create a categorical variable indicating distance to HTR (high traffic road more than 250 m, between 150 and 250 m, between 100 and 150 m, between 50 and 100 m, and less than 50 m away). We calculated a five category variable of total length of high traffic road segments within a 150 m buffer zone as the quartiles of the sum of segments' lengths within the 150 meters buffer (none, low <166 m, medium 166-266 m, high 266-323 m, very high 323-1445 m).

We collected and stored all geographical variables using ArcGis 9.1 (ESRI, Redlands, California, USA). We used the Word Geodetic System of 1984 with the Universal Transverse Mercator 33N as the coordinate system and map projection.

### Baseline health status: individual morbidity history

In Italy there is a National Health Service that provides medical care to all the population. The morbidity history of the study population was based on data from the Health Information System of the Lazio region, where Rome is located. The regional Health Information System collects individual discharge records from all hospitals, both public and private. All the records are linkable using a unique identifier, but privacy protection is assured from strict management rules. Discharge records are routinely collected and contain: patient demographic data, admission and discharge dates, up to six discharge diagnoses (International Classification of Disease, 9^th ^revision, Clinical Modification [ICD-9-CM]), medical procedures or surgical interventions (up to six), and status at discharge (alive, dead, transferred to other hospital). In order to describe the baseline health characteristics of the cohort, we used hospital discharges from 1996 to 2001 to identify those individuals who had at least one hospitalisation. We considered hospitalisations for all causes excluding accidents, those with a principal diagnosis of cardiovascular diseases (ICD-9-CM: 390-459), with principal or secondary diagnoses of hypertension (ICD-9-CM: 401-405), with principal diagnosis of ischemic heart disease (ICD-9-CM: 410-414), congestive heart failure (ICD-9-CM: 398.91, 402.01, 402.11, 402.91, 404.01, 404.03, 404.11, 404.13, 404.91, 404.93, 425.4-425.9, 428), peripheral vascular disorders (ICD-9-CM: 093.0, 437.3, 440, 441, 443, 447.1, 557.1, 557.9, V43.4), arrhythmia (ICD-9-CM: 426.0, 426.13, 426.7, 426.9, 426.10, 426.12, 427.0-427.4, 427.6-427.9, 785.0, 996.01, 996.04, V45.0, V53.3), cerebrovascular disease (ICD-9-CM: 430-438); for cancer (ICD-9-CM: 140-239); diabetes (ICD-9-CM: 250), and chronic obstructive pulmonary disease (ICD-9-CM: 490-492, 494, 496).

### Statistical analysis

We studied the association of traffic variables with age and socioeconomic position. We considered two SEP indices (area-based SEP and individual level of education) and three traffic measures (a binary variable to identify who lives within 50 meters of an HTR, the distance from HTR, and the traffic density within 150 m).

We used logistic regression to evaluate the associations with living close (50 m) to an HTR (Odds ratios, OR, with 95 percent confidence intervals were calculated). We log-transformed both the distance from an HTR and the traffic density to obtain two normally distributed variables, and we used them as dependent variables in a multivariate linear regression analysis. For both logistic and linear regression models, the independent variables were age (0-17, 18-34, 35-64, 65-74, 75+ years), education, and area-based SEP. In the presentation of the results of the linear regression models, we calculated the exponential function of the regression coefficients in order to estimate the ratio of the dependent variable in the specific subgroup compared to the reference group (geometric mean ratio, GMR). We calculated 95 percent confidence intervals of the GMR. To take into account the clustering of the subjects within census blocks, we performed all multiple regression analyses with robust variance estimate.

As a final step, we performed a stratified analysis dividing the entire population by area of residence (inside and outside the central railway ring) to better understand the relationship between age, small area SEP, and education with traffic exposure.

## Results

The total population of Rome in October 2001 was 2.56 million (2001 Census) people. For 2,118,670 residents (84%) we had information on the address of residence and on individual socioeconomic position. We selected only those individuals whose address was matched and geocoded by the Tele Atlas system with optimal quality, resulting in 1,898,898 subjects that were considered in this study. There were no significant differences between subjects included and excluded from the analysis for age, and gender.

Table 1 shows the socio-demographic characteristics of the study population in five age groups. Fifteen percent of the study population was under 18 years of age, while 19% was aged 65 or more. The population by age group was not evenly distributed across the area-based socioeconomic (SEP) index: the youngest groups were more likely to live in low SEP areas compared to the oldest group. Also level of education increased with age.

**Table 1:** Socio-demographic characteristics of study population by age group

	Age (years)					
	0-17	18-34	35-64	65-74	75+	Total
**N**	289,539	420,169	821,153	214,332	153,705	1,898,898
*(%males)*	51.4	49.6	46.9	44.5	37.2	47.1
						
**Area based SEP**						
High	18.2	17.4	19.6	19.9	23.8	19.3
Medium-high	18.6	18.9	20.3	20.4	23.0	19.9
Medium	18.6	19.3	20.0	20.5	20.8	19.7
Medium-low	21.2	21.4	20.4	20.5	18.1	20.6
Low	23.5	23.0	19.8	18.7	14.4	20.5
						
Education*						
University	0.0	13.9	18.5	10.9	11.4	13.2
High school	1.5	59.5	37.5	17.9	16.5	32.9
Secondary school	59.4	26.2	42.4	62.5	59.3	42.2
Primary school	39.1	0.4	1.6	8.7	12.9	6.9
						
**Employment status†**						
Employed	0.7	51.4	61.5	5.5	1.1	38.7
Looking for first employment	3.5	10.3	0.9	0.0	0.0	2.8
Unemployed	1.0	9.2	5.1	0.2	0.0	4.3
Student	94.3	22.3	0.2	0.0	0.0	7.5
Housewife	0.1	4.3	18.7	29.6	30.9	14.9
Retired	0.0	0.1	10.8	58.4	57.9	16.0
Military or civil service	0.0	0.6	0.0	0.0	0.0	0.1
Unable to work	0.1	0.5	1.1	1.4	2.4	0.9
Other	0.4	1.2	1.8	4.8	7.8	2.2
						
**Occupation‡**						
Non manual	6.7	55.8	64.7	68.0	73.7	62.1
*Managers*	*1.0*	*5.9*	*12.1*	*25.6*	*24.7*	*10.5*
*Highly-skilled*	*0.0*	*12.3*	*18.2*	*28.4*	*38.4*	*16.6*
*Medium-skilled*	*2.4*	*25.0*	*21.6*	*10.8*	*9.5*	*22.4*
*Unskilled*	*3.3*	*12.6*	*12.8*	*3.1*	*1.0*	*12.6*
Manual	93.3	41.8	33.4	31.8	26.3	35.9
*Services*	*35.2*	*19.7*	*11.4*	*13.0*	*12.6*	*13.9*
*Highly-skilled*	*24.5*	*8.1*	*7.4*	*6.7*	*3.5*	*7.6*
*Medium-skilled*	*7.1*	*4.5*	*3.8*	*2.4*	*1.9*	*4.0*
*Unskilled*	*26.0*	*9.3*	*10.3*	*7.6*	*6.2*	*10.0*
*Farmer*	*0.5*	*0.3*	*0.4*	*2.0*	*2.1*	*0.4*
Military forces	0.0	2.3	1.9	0.2	0.0	2.0

*(> = 6 year olds)

†(> = 15 year olds)

‡(on those employed)

Descriptive data of exposure to traffic by age are reported in table 2. The subjects' homes were an average of 272 meters (standard deviation, SD, 459) from a high traffic road. A total of 34% of the population lived further than 250 meters from an HTR, while 45% had an HTR within 150 meters, and 17% lived closer than 50 m from an HTR. The daily average traffic count from the closest HTR (within 150 m from home) was 21,533 vehicles (SD 15,094). The majority of subjects with an HTR within 150 meters had traffic counts ranging from 10,000 to 15,000 while a minority had more than 30,000. For residents living closer than 150 meters from an HTR, the mean traffic density was 107 vehicle meters of HTR/m^2 ^(SD 88).

**Table 2:** Environmental characteristics of the study population by age group.

	Age (years)					
	0-17	18-34	35-64	65-74	75+	Total
**N**	289,539	420,169	821,153	214,332	153,705	1,898,898
						
**Distance from HTR** mean (sd) %	302 (506)	286 (480)	274 (457)	241 (420)	208 (340)	272 (459)
> = 250	37.4	35.6	34.4	30.2	25.9	34.0
[150-250)	20.5	20.2	20.6	21.4	21.5	20.7
[100-150)	14.0	14.3	14.6	15.4	16.2	14.6
[50-100)	12.9	13.5	13.8	14.7	16.0	13.9
< = 50	15.3	16.4	16.6	18.3	20.4	16.9
						
**Meters of HTR within 150 m from home**						
mean (sd) for those who live at < = 150 m from HTR %	268 (159)	271 (161)	272 (161)	276 (163)	282 (166)	272 (161)
living at > = 150 m from HTR	57.9	55.8	55.1	51.6	47.4	54.6
1st quartile (<166 m)	10.8	11.2	11.3	11.7	12.2	11.3
2nd quartile (166-266 m)	10.7	11.1	11.3	11.9	12.9	11.3
3rd quartile (266-323 m)	10.6	11.0	11.2	12.3	13.1	11.3
4th quartile (323-1445 m)	10.0	11.0	11.1	12.5	14.4	11.3
						
**Daily average traffic count on the closest HTR within 150 m**						
mean (sd) for those who live at < = 150 m from HTR %	21686 (14272)	21832 (14255)	21774 (14006)	21975 (13497)	21920 (12776)	21533 (15094)
living at > = 150 m from HTR	57.9	55.8	55.1	51.6	47.4	54.6
(10000-15000)	17.9	18.4	18.7	19.3	20.7	18.8
[15000-20000)	7.9	8.5	8.5	9.0	9.6	8.6
[20000-30000)	7.9	8.3	8.5	9.6	10.7	8.7
> = 30000	8.4	9.0	9.2	10.4	11.6	9.4
						
**Traffic density from HTR within 150 m (vehicle meters of HTR/m^2^)**						
mean (sd) for those who live at < = 150 m from HTR %	105 (91)	107 (91)	106 (88)	109 (85)	111 (81)	107 (88)
living at > = 150 m from HTR	57.9	55.8	55.1	51.6	47.4	54.6
1st quartile (< = 51)	11.4	11.3	11.4	11.2	11.4	11.3
2nd quartile (52-82)	10.5	11.1	11.3	12.1	12.8	11.3
3rd quartile (83-137)	10.3	10.9	11.2	12.4	13.9	11.3
4th quartile (137-1257)	9.9	10.9	11.1	12.7	14.5	11.3

Table 3 shows the percentages of subjects who were hospitalised for specific diseases during the period 1996-2001, before the enrolment in the study. Overall hospitalisations include births but not accidents. Twenty-nine percent of the study population had been admitted to hospital in the five years before enrolment (5% for cardiovascular causes). The percentage with a previous hospitalisation increases with age, reaching 16.5% for hypertension in the 75+ age group.

**Table 3:** Percentages of subjects who were hospitalised for specific diseases during 1996 and 2001, before the enrolment in study by age group

	**Age (years)**					
	**0-17**	**18-34**	**35-64**	**65-74**	**75+**	**Total**
	
**Subjects**	289,539	420,169	821,153	214,332	153,705	1,898,898
**Subjects hospitalized %**	58,304	94,211	226,109	87,416	74,890	540,930
Non accidental causes	20.1	22.4	27.5	40.8	48.7	28.5
Cardiovascular causes	0.49	1.50	4.27	11.59	16.24	4.88
*Hypertension*	*0.05*	*0.12*	*2.52*	*11.07*	*16.52*	*3.71*
*Ischemic Hearth Disease*	*0.01*	*0.02*	*1.13*	*5.33*	*8.36*	*1.77*
*Congestive Heart Failure*	*0.01*	*0.01*	*0.25*	*1.67*	*4.01*	*0.63*
*Peripheral Vascular Disorders*	*0.01*	*0.03*	*0.30*	*1.98*	*3.59*	*0.65*
*Arrhytmia*	*0.18*	*0.14*	*0.69*	*3.50*	*7.26*	*1.34*
*Cerebrovascular Disease*	*0.02*	*0.05*	*0.53*	*3.36*	*7.33*	*1.21*
Cancer	0.06	0.24	1.60	4.70	5.40	1.72
Diabetes	0.06	0.11	0.91	4.17	5.72	1.36
Chronic Pulmonary Disease	0.90	0.17	0.68	3.87	7.03	1.47

Table 4 shows the association (odds ratios and geometric mean ratios) of age, area-based socioeconomic position, and level of education with the three measures of traffic exposure (living 50 meters from an HTR, distance from HTR and traffic density within 150 m from home). The table reports odds ratios or GMR adjusted only for sex and age group and adjusted for all the factors in the table. We observed that older people were more likely to live in high-traffic areas and the association was confirmed for the three traffic indicators when adjustment was made for the SEP variables. Twenty-one percent of subjects living in medium SEP census blocks had a high traffic road within 50 meters of home, versus 18% of high area-based SEP, and 12% of low-area based SEP. The results of the multivariate analyses indicated that those belonging to the medium area-based SEP index level were the most likely to have heavy traffic within 50 meters. In addition, those living in the lowest two area-based SEP categories were less likely to live close to an HTR compared to others even when age group and educational level were taken into account. The results were similar when we used the continuous variables indicating distance from HTR and traffic density as the exposure measures. All three indicators of traffic exposure showed that less educated subjects were less exposed to traffic than more educated residents; these associations were attenuated when area-based SEP was taken into account.

**Table 4:** Association of socioeconomic position (SEP) with traffic exposure

	Living at < = 50 m from HTR					Distance from HTR				Traffic density from HTR within 150 m from home			
			
	%	OR^†^	95% CI	OR^‡^	95% CI	GMR^†^	95% CI	GMR^‡^	95% CI	GMR^†^	95% CI	GMR^‡^	95% CI
**Age group**													
0-17	15.3	1.00		1.00		1.00		1.00		1.00		1.00	
18-34	16.4	1.09	1.06 - 1.11	1.05	1.02 - 1.09	0.94	0.92 - 0.95	0.97	0.95 - 0.99	1.03	1.02 - 1.04	1.02	1.01 - 1.04
35-64	16.6	1.10	1.08 - 1.13	1.05	1.02 - 1.08	0.91	0.90 - 0.93	0.96	0.94 - 0.98	1.03	1.02 - 1.04	1.02	1.01 - 1.03
65-74	18.3	1.24	1.19 - 1.29	1.19	1.14 - 1.24	0.81	0.79 - 0.83	0.85	0.82 - 0.87	1.07	1.05 - 1.08	1.06	1.04 - 1.08
75+	20.4	1.41	1.35 - 1.48	1.33	1.27 - 1.39	0.71	0.69 - 0.74	0.77	0.74 - 0.79	1.10	1.08 - 1.12	1.09	1.07 - 1.11
													
**Area based SEP**			-		- -		-		-				
High	18.1	1.00	-	1.00		1.00	-		-	1.00		1.00	
Medium-high	19.0	1.07	0.91 - 1.26	1.08	0.92 - 1.27	0.94	0.84 - 1.05	0.93	0.83 - 1.04	1.06	0.98 - 1.14	1.06	0.99 - 1.14
Medium	20.8	1.20	1.01 - 1.41	1.21	1.03 - 1.43	0.93	0.82 - 1.04	0.92	0.82 - 1.03	1.11	1.03 - 1.19	1.11	1.04 - 1.20
Medium-low	15.0	0.81	0.68 - 0.96	0.82	0.69 - 0.98	1.31	1.16 - 1.47	1.28	1.14 - 1.44	1.01	0.94 - 1.09	1.02	0.94 - 1.09
Low	11.7	0.61	0.50 - 0.74	0.62	0.51 - 0.76	1.60	1.41 - 1.81	1.57	1.38 - 1.77	0.86	0.79 - 0.93	0.86	0.79 - 0.94
													
Education*			-		-		-		-				
University	18.6	1.00	-	1.00	-	1.00	-		-	1.00		1.00	
High school	17.3	0.93	0.90 - 0.96	0.97	0.94 - 0.99	1.10	1.08 - 1.12	1.05	1.03 - 1.07	0.98	0.96 - 0.99	0.98	0.97 - 0.99
Secondary school	16.3	0.83	0.78 - 0.88	0.95	0.91 - 0.99	1.24	1.19 - 1.30	1.06	1.04 - 1.09	0.95	0.92 - 0.97	0.98	0.96 - 1.00
Primary school	15.4	0.76	0.71 - 0.82	0.91	0.86 - 0.95	1.34	1.27 - 1.41	1.11	1.07 - 1.14	0.94	0.91 - 0.97	0.98	0.96 - 1.00

*(> = 6 year olds)

† multiple regression models including sex, and age group

‡ multiple regression models including sex, age group, area-based SEP, and education

Table 5 shows the association between SEP and traffic exposure by area of residence (inside and outside the central railway ring, corresponding to 298,326 and 1,600,572 cohort members, respectively). The central part of Rome, delimited by the railway ring and corresponding to the historical centre, is characterized by a resident population older (65+ years: 24.3% versus 18.5%) and with a higher socioeconomic position than the rest of the city (both at individual and area level; e.g. for high area-based SEP: 35.9% versus 16.4%; for low area-based SEP: 3% versus 23.7%). In both the areas there is evidence of association between older age and living in proximity of an HTR, however, the association between traffic exposure and SEP had a different sign in the two areas. In the city centre where traffic is higher and 25% of the residents lives close to HTR, less affluent and less educated people tend live closer to HTR than more affluent and highly educated people, the opposite is seen in the rest of the city (where traffic is lower and only 15% lives close to HTR) in agreement with the overall results.

**Table 5:** Association of socioeconomic position (SEP) with traffic exposure by area of residence

	Residents in the railway ring (N = 298,326)						Residents outside the railway ring (N = 1,600,572)					
		
		Living at < = 50 m from HTR						Living at < = 50 m from HTR				
				
	N	%	OR^†^	95% CI	OR^‡^	95% CI	N	%	OR^†^	95% CI	OR^‡^	95% CI
**Age group**												
0-17	39,515	23.6	1.00		1.00		250,024	14.0	1.00		1.00	
18-34	58,731	25.4	1.10	1.05 - 1.15	1.18	1.10 - 1.27	361,438	14.9	1.08	1.05 - 1.11	1.05	1.01 - 1.09
35-64	128,539	24.6	1.05	1.02 - 1.09	1.15	1.07 - 1.22	692,614	15.1	1.10	1.07 - 1.12	1.05	1.02 - 1.08
65-74	36,099	25.2	1.09	1.04 - 1.15	1.14	1.06 - 1.22	178,233	16.9	1.25	1.19 - 1.31	1.21	1.15 - 1.27
75+	35,442	25.2	1.09	1.04 - 1.14	1.15	1.08 - 1.22	118,263	18.9	1.43	1.36 - 1.51	1.36	1.29 - 1.43
												
**Area based SEP**												
High	107,244	21.8	1.00		1.00		258,421	16.5	1.00		1.00	
Medium-high	96,397	24.5	1.17	0.91 - 1.49	1.16	0.91 - 1.48	282,154	17.2	1.05	0.86 - 1.29	1.06	0.87 - 1.30
Medium	59,480	28.5	1.43	1.09 - 1.89	1.41	1.07 - 1.86	315,283	19.4	1.22	0.99 - 1.49	1.23	1.00 - 1.51
Medium-low	26,347	27.2	1.34	0.90 - 1.99	1.30	0.88 - 1.93	364,457	14.1	0.84	0.68 - 1.03	0.85	0.69 - 1.05
Low	8,858	31.5	1.65	0.74 - 3.66	1.60	0.72 - 3.59	380,250	11.2	0.65	0.52 - 0.81	0.66	0.53 - 0.82
												
Education*												
University	75,040	22.5	1.00		1.00		175,926	17.0	1.00		1.00	
High school	103,950	25.2	1.15	1.10 - 1.21	1.14	1.09 - 1.19	520,729	15.8	0.93	0.90 - 0.97	0.96	0.93 - 0.99
Secondary school	90,623	26.5	1.29	1.18 - 1.42	1.24	1.14 - 1.34	710,587	15.0	0.84	0.78 - 0.90	0.95	0.90 - 0.99
Primary school	16,179	24.1	1.24	1.12 - 1.37	1.17	1.07 - 1.28	114,722	14.2	0.77	0.71 - 0.84	0.90	0.85 - 0.96

*(> = 6 year olds)

† multiple regression models including sex, and age group

‡ multiple regression models including sex, age group, area-based SEP, and education

## Discussion

The study clearly shows that a large fraction of the Rome population is exposed to traffic at home. This is not surprising when one considers that out of a population of 2.6 million inhabitants, almost 2.3 million vehicles were circulating in the city in 2001, including moped/motorcycles, and commercial vehicles. The public health problem of traffic is amplified as one considers that in 2001 a large proportion of the vehicles were in the high emissions categories (EURO 0 and EURO 1) and that 17.4% of them were diesel-powered vehicles. Exposure to traffic is not evenly distributed in the city: we found that all the measures of traffic exposure were higher in the elderly population than in younger groups. In general, traffic was not higher in the poorest sector of the population, on the contrary, low SEP was associated with greater distance from HTRs and with lower traffic density within 150 m from residence. However, when we restricted the analysis to the city centre, higher exposures to traffic were found among subjects in the low socioeconomic groups. In any case it appeared that area level more than individual level drives the association between SEP and traffic exposure.

The explanation for the traffic exposure and age and SEP relationships is clearly related to the urbanization history in Rome with the consequence that people of medium-high social class are located in the more central and prestigious areas of the city where there are more high traffic roads. The prices of dwellings in the city centre are much higher than in the suburbs and the poor air quality is rewarded by prestigious buildings, good quality of public transportation, and access to several facilities. This clearly explains why elderly are more exposed to traffic air pollution: in the past it was still affordable for young couples to buy a flat in the city centre, but now the value of dwellings is so high compared to the suburbs that in the last decades young new families have tended to live in the periphery. The same reasons explain the different socioeconomic distribution of population by area of the city, new residents in the city centre tend to be very well-off whereas less advantaged people move from the centre to the suburbs for economic reasons. It is interesting, however, that restricting the analysis to the city centre, the relationship between SEP and traffic exposure changes, with low exposures in residents better educated or who live in a high SEP area, suggesting that environmental inequalities do exist in the central area.

As indicated before, the large number of people residing close to busy roads in Rome underlines the need for a public health attention to the issue. Unfortunately, there are few comparable measures from other cities in the world to evaluate whether other places have similar distribution values to what we found in Rome. In the Netherland cohort study, almost 5 percent of the study members lived within 50 meters of a busy road, whereas it has been reported that the proportion of the population living within 50 meters from a major road in Los Angeles and Toronto is approximately 32% [6,14]. A meta-analysis of the available evidence indicates that for most pollutants there is a distant decay gradient in the range of 100-400 meters from the source and therefore the population with direct exposure to traffic-related pollution is even higher [33].

Our results on the association between SEP and traffic exposure were similar to those from a study in the Netherlands [5], but in contrast with the results from studies on environmental equity from other countries, especially the US [15,34-36]. However a study by Buzzelli and Jerrett found that in Toronto racial minority groups were less exposed to air pollution than other groups, and that dwelling values predicted total suspended particulates levels in Canada [37].

Most of the early evidence that people of lower socioeconomic status are more exposed to air pollution comes from studies that have considered point sources, mainly industrial settlements [14]. On the other hand, the recent literature available on exposure to traffic is more limited. Comparability is also difficult given the use of diverse measures of exposure to air pollution, and different measures of socioeconomic position. In the studies conducted in the US, it is taken for granted that disadvantaged groups of the population live in the worst environmental conditions including high exposure to outdoor air pollution [17]. It should be recognized that the literature on the issue is still limited and additional observations are needed from different parts of the world. Our finding in Rome is not new. In a previous investigation on short-term effects of PM_10_ in the city, we pointed out that emissions of PM, CO, NOx, and benzene were higher in areas of higher socioeconomic status [25]. In addition, in a large cross-sectional study of adults, GIS indices of exposure to traffic and estimated NO_2 _level at the residential address from a land use regression model were directly linked with socioeconomic position [13].

Despite the socioeconomic gradient of exposure, the short-term mortality effects of PM_10 _in Rome were larger in the low socioeconomic groups perhaps as a result of a different susceptibility level of poor people rather than to different proximity to traffic [25]. On the other hand, the fact that higher socioeconomic groups live in high traffic areas does not necessarily mean that they are more exposed than underprivileged groups are. More advantaged people work in air-filtered office buildings than outside, they use private transportation more than public transportation, they are more likely to live closer to their work places, they are more likely to spend the week-ends outside the city than low socioeconomic groups. Clearly, investigations on personal exposure to air pollutants are needed to better qualify the issue.

Important new evidence has been accumulating on the effects of long-term exposure to traffic-related air pollutants. Although the relationship between traffic-related air pollutants and cardiovascular mortality and morbidity has not been clearly defined yet, it is clear that particulate matter has an effect on the cardiorespiratory system [14,38]. An important study on long-term effects of PM_2.5 _on the cardiovascular system was conducted in the US [39]. The follow-up included more than 65,000 postmenopausal women (Women Health Initiative) without previous cardiovascular disease in 36 US metropolitan areas from 1994 to 1998. Each 10 μg/m^3 ^increase in PM_2.5 _was associated with a 24% increase in the risk of a cardiovascular event and a 76% increase in the risk of death from cardiovascular disease. A report from Oslo has linked long-term exposure to traffic-related pollutants to cause-specific mortality [40]. A consistent effect on all causes of death was found for both sexes and age groups; the effects were particularly strong for COPD. The study shows that people with COPD disease and the elderly seem to be affected by air pollution at lower levels than the general population. Important results are available for myocardial infarction as its occurrence [9,41] and survival have a strong links with particulate matter exposure [42].

This study has some limitations. First, we used proxy measure of exposure at residence rather individual measures of exposure to traffic related pollutants. Second, we used the interpolation method for geocoding instead of a building/parcel method, and this could result in an inaccurate exposure characterization, especially in the periphery of Rome where street segments are longer than in the centre. Finally, when we geocoded the addresses of participants living in a multi-storey building, we assigned the distance to the front door to all residents in the building, irrespective to their real position.

The results of our investigation with respect to the history of previous hospitalization should be interpreted for descriptive purposes only. They are useful to evaluate the specific vulnerability of the population in relation to the environmental exposure and they indicate that from a public health point of view there is a sub-population that is more susceptible that needs to be protected in a more intensive way.

## Conclusions

We have shown that living nearby traffic is a widespread phenomenon in Rome, and that people who live close to traffic are older, better educated, and reside in higher socioeconomic areas than those who live far. The study population will be followed to better understand the potential health effects of traffic-related air pollution exposure.

## Abbreviations

HTR: high traffic road; SEP: socioeconomic position; GIS: geographic information system; PM: ambient particulate matter; SD: standard deviation; OR: odds ratio; GMR: geometric mean ratio; CI: confidence interval; CVD: cardiovascular diseases; COPD: chronic obstructive pulmonary disease; CO: carbon monoxide; NOx: nitrogen oxide.

## Competing interests

The authors declare that they have no competing interests.

## Authors' contributions

GC participated in the design of the study, performed the analysis, and drafted the manuscript. CB performed all GIS analysis. VR provided data on traffic and helped to discuss the results. ED provided GIS data, and helped in interpreting the results. CAP participated in coordination and interpretation. All authors read and approved the manuscript. FF participated in the design and coordination of the study, and helped to draft the manuscript.
